# PINK1–Associated Mitophagy Protects Against Cisplatin–Induced Ototoxicity by Regulating the Subcellular Distribution of ACSL4

**DOI:** 10.3390/cells15181657

**Published:** 2026-09-14

**Authors:** Ting Li, Wenting Yu, Huanzhi Wan, Xuan Yu, Xi Lian, Ziqi Zhou, Shimin Zong, Hongjun Xiao

**Affiliations:** 1 Department of Otorhinolaryngology–Head and Neck Surgery, Union Hospital, Tongji Medical College, Huazhong University of Science and Technology, Wuhan 430022, China; 2 Institute of Otorhinolaryngology–Head and Neck Surgery, Tongji Medical College, Huazhong University of Science and Technology, Wuhan 430022, China; 3 Hubei Province Clinical Research Center for Deafness and Vertigo, Wuhan 430022, China

**Keywords:** cisplatin, ototoxicity, PINK1, mitophagy, ACSL4, ferroptosis

## Abstract

Cisplatin–induced ototoxicity is a significant clinical issue linked to ferroptosis and mitophagy dysfunction. This study investigated how mitophagy regulates ferroptosis in cisplatin–induced hearing loss using cultured primary rat cochlear marginal cells in vitro and a C57BL/6J mouse model of cisplatin–induced ototoxicity in vivo. Cisplatin suppressed the PTEN–induced putative kinase 1 (PINK1) pathway, impaired mitophagy, and triggered ferroptosis characterized by iron overload, lipid peroxidation, increased acyl–CoA synthetase long–chain family member 4 (ACSL4), and decreased glutathione peroxidase 4. Pharmacological activation of mitophagy or PINK1 overexpression alleviated ferroptosis in vitro, while deferoxamine treatment reduced cisplatin–induced hearing loss and blood–labyrinth barrier damage in vivo. Mechanistically, restoration of mitophagy was associated with increased mitochondrial targeting and reduced cytosolic accumulation of ACSL4, potentially through mitophagic clearance, thereby suppressing ferroptosis. These findings support a model in which impaired PINK1–associated mitophagy contributes to cytosolic ACSL4 accumulation and ferroptotic injury. Modulating mitophagy and ACSL4 subcellular distribution may represent a potential strategy for mitigating cisplatin–induced ototoxicity.

## 1. Introduction

Cisplatin (Cis) is one of the most widely used chemotherapeutic agents in clinical practice and shows remarkable efficacy against a variety of solid tumors, including lung cancer, ovarian cancer, and head and neck malignancies [1,2]. However, its clinical application is severely limited by irreversible ototoxicity, which occurs in approximately 20% to 70% of treated patients [3,4,5]. Cisplatin–induced ototoxicity is primarily characterized by high–frequency sensorineural hearing loss, accompanied by cochlear structural and pathological changes such as damage to the stria vascularis (SV) and disruption of the blood–labyrinth barrier (BLB) [5,6,7]. Marginal cells (MCs) of the cochlea, being a core component of the SV, play a critical role in maintaining endolymphatic homeostasis. Dysfunction of these cells is closely linked to cisplatin–induced hearing impairment [7,8,9,10]. Despite decades of research, the molecular mechanisms underlying cisplatin–induced ototoxicity remain incompletely understood, and effective clinical strategies for its prevention or treatment are still lacking [5,11,12].

In recent years, accumulating evidence has established ferroptosis, a novel form of regulated cell death characterized by iron overload and excessive lipid peroxidation, as a key pathological driver underlying cisplatin–induced ototoxicity, leading to damage in various cochlear structures including spiral ganglion neurons (SGNs), hair cells (HCs), synapses, and the SV [6,13,14,15].

Mitochondria, serving as the cellular “powerhouses,” are major sites of reactive oxygen species (ROS) production and a primary target of cisplatin–induced damage [12,16,17]. Mitophagy, a selective form of autophagy, serves to maintain mitochondrial homeostasis through the clearance of damaged mitochondria and is a crucial cellular protective mechanism under stress [18,19]. The PTEN–induced putative kinase 1 (PINK1)/Parkin (PARK2) pathway represents the best–characterized axis regulating mitophagy: under physiological conditions, PINK1 is constitutively degraded; upon mitochondrial damage, it stabilizes on the outer mitochondrial membrane to recruit and activate the E3 ubiquitin ligase Parkin, which tags damaged mitochondria for autophagic degradation, thereby restoring homeostasis [20,21]. While mitophagy’s role as a key homeostatic mechanism has garnered some support in the context of otoprotection, direct evidence specifically linking it to cisplatin–induced ototoxicity is still scarce [16,22,23].

Currently, the relationship between mitophagy and ferroptosis in the context of cisplatin–induced injury remains elusive. On one hand, inhibition of mitophagy leads to the accumulation of damaged mitochondria, resulting in the cytosolic release of ROS and ferrous iron (Fe^2+^), which exacerbates lipid peroxidation and facilitates ferroptosis [24,25,26,27]. On the other hand, enhanced mitophagy has also been reported to increase the release of mitochondrial labile iron and ROS, a process termed “mitophagy–dependent ferroptosis” [24,25,26,27]. These apparently contradictory findings highlight the potential dual role of mitophagy in modulating ferroptosis.

Ferroptosis is tightly controlled by a set of core regulators, among which acyl–CoA synthetase long–chain family member 4 (ACSL4) is a pivotal driver. ACSL4 catalyzes the biosynthesis of polyunsaturated fatty acid phospholipids, thereby providing the essential substrates for lipid peroxidation and promoting ferroptosis [28,29]. It is thus plausible that ACSL4 contributes to cisplatin–induced ototoxicity by regulating ferroptosis. However, direct experimental evidence regarding the effect of cisplatin on the expression, activity, and functional role of ACSL4 in the cochlea is lacking. Whether cisplatin triggers cochlear cell ferroptosis through ACSL4 awaits validation. More importantly, the upstream signaling pathways that govern ferroptosis initiation and execution in cisplatin ototoxicity remain largely unknown. Notably, recent studies have revealed a dynamic shuttling of ferroptosis–related molecules, including ACSL4 and glutathione peroxidase 4 (GPX4), between the cytoplasm and mitochondria [30,31,32]. Yet, whether and how this subcellular localization mechanism participates in cisplatin–induced ototoxicity is still an open question.

Therefore, this study investigated the relationship between PINK1–associated mitophagy and ferroptosis in cisplatin–induced ototoxicity, with a focus on cochlear MCs. Our results showed that cisplatin-induced ferroptotic injury and hearing loss were accompanied by impaired PINK1-associated mitophagy. Restoration of mitophagy attenuated ferroptotic injury and was associated with increased mitochondrial targeting and reduced cytosolic accumulation of ACSL4, potentially through mitophagic clearance. These findings support a model in which PINK1-associated mitophagy regulates ferroptosis by modulating the subcellular distribution and clearance of ACSL4, and suggest that enhancing mitophagy may represent a potential strategy for mitigating cisplatin-induced ototoxicity.

## 2. Materials and Methods

### 2.1. Animals

Six-week-old male C57BL/6J mice, weighing 17–19 g, and postnatal day 1 Sprague-Dawley (SD) rats were purchased from Hubei Biont Biological Technology Co., Ltd. (Wuhan, China). The animals were housed under specific pathogen-free (SPF) conditions in a controlled environment (temperature: 21 ± 1 °C; humidity: 55–60%; 12 h light/dark cycle). All experimental protocols were approved by the Animal Welfare Committee of Tongji Medical College, Huazhong University of Science and Technology (Ethical Approval No.: 2024-4974) and were performed in compliance with the Guide for the Care and Use of Laboratory Animals (NIH Publication No. 8023, revised 1978).

C57BL/6J mice were administered cisplatin (12 mg/kg/day, Cat. #C295225, Aladdin, Shanghai, China) [3] and deferoxamine (DFO; 100 mg/kg/day, Cat. #HY-B1625, MedChemExpress, Monmouth Junction, NJ, USA) via intraperitoneal injection. Control mice received an equal volume of sterile saline.

### 2.2. Auditory Brainstem Response (ABR) Threshold Measurement

ABR thresholds were measured in C57BL/6J mice one day before the initial DFO or saline injection and 3 days after the final injection [3]. Before testing, all tympanic membranes were examined via otoscopy to confirm they were dry and showed no evidence of infection or effusion. Mice were anesthetized by intraperitoneal injection of 2.5% tribromoethanol (0.2 mL/10 g body weight, Cat. #75-80-9, Meilunbio, Dalian, China) and positioned on a thermostatically controlled heating pad in a prone position. Subdermal electrodes were placed at the vertex, near the tested ear, and near the contralateral ear as the recording, reference, and ground electrodes, respectively. Click stimuli and tone-burst stimuli at 8, 12, 16, 20, 24, 28, and 32 kHz were generated and delivered using a Tucker–Davis Technologies system (Alachua, FL, USA). A free-field speaker was placed 3 cm directly in front of each mouse’s nose to ensure equal sound stimulation of both ears. The ABR threshold was defined as the lowest stimulus intensity that elicited a reproducible waveform.

### 2.3. Histological Analysis (Hematoxylin–Eosin [H&E] Staining and Immunohistochemistry [IHC])

#### 2.3.1. H&E Staining

For histological analysis, paraffin-embedded tissue sections were cut, dewaxed in xylene, and rehydrated through a graded ethanol series. Sections were stained with Mayer’s hematoxylin for 5 min, rinsed under running tap water, differentiated in 1% acid-alcohol for a few seconds, and blued in running water. Subsequently, sections were counterstained with eosin for 2 min, dehydrated through an ascending ethanol series, cleared in xylene, and cover-slipped with a neutral mounting medium.

#### 2.3.2. IHC

For IHC, processed sections were subjected to heat-mediated antigen retrieval in citrate buffer (pH 6.0). Endogenous peroxidase activity was quenched by incubation with 3% hydrogen peroxide for 15 min at room temperature. Non-specific binding was blocked by incubation with 10% normal goat serum in phosphate-buffered saline (PBS) for 30 min at room temperature. Sections were then incubated overnight at 4 °C with primary antibodies (details provided in Supplementary Materials [Table S1]). Following three washes in PBS, sections were incubated with horseradish peroxidase (HRP)-conjugated secondary antibodies for 1 h at room temperature. Immunoreactivity was visualized using a 3,3′-diaminobenzidine (DAB) substrate kit, and nuclei were counterstained with hematoxylin. Finally, sections were dehydrated, cleared, and mounted as described for H&E staining. Whole-slide images were acquired using a high-resolution slide scanner.

### 2.4. Isolation and Culture of Primary MCs

Primary MCs were isolated from the cochlear SV of postnatal day 1 SD rats and cultured as described below. These cultured primary rat MCs were used in all subsequent in vitro experiments. Rats were anesthetized by hypothermia and euthanized, after which bilateral cochleae were rapidly dissected and placed in ice-cold Hanks’ Balanced Salt Solution (HBSS). Under a dissecting microscope, the SV was carefully isolated. The tissue was then enzymatically digested using 0.1% collagenase type II (Sigma-Aldrich, St. Louis, MO, USA) at 37 °C for 30 min [8]. Following digestion, the cell suspension was centrifuged at 1000× *g* for 5 min. The resulting pellet was resuspended in EpiCM-animal epithelial cell medium (ScienCell, Carlsbad, CA, USA) and seeded onto culture plates. Cells were maintained at 37 °C in a humidified 5% CO_2_ incubator.

For pharmacological treatments, cells were exposed to cisplatin (10 μM; Plant Pharmaceutical Industry Co., Ltd., Kunming, China) for 24 h to induce injury. To inhibit ferroptosis, cells were pre-treated with Ferrostatin-1 (Fer-1; 1 μM; MedChemExpress, Cat# HY-100579, Monmouth Junction, NJ, USA) for 24 h prior to cisplatin administration. To induce mitophagy, cells were co-treated with the protonophore carbonyl cyanide m-chlorophenyl hydrazone (CCCP; 10 μM; MedChemExpress, Cat# HY-100941, Monmouth Junction, NJ, USA) and cisplatin.

### 2.5. Cell Viability Assay

Cell viability was assessed using a Cell Counting Kit-8 (CCK-8, Biosharp, Beijing, China, Cat# BS350B). Following the specified pharmacological treatments, 10 μL of CCK-8 reagent was added to each well of a 96-well culture plate. The plate was then incubated for 2 h under standard culture conditions (37 °C, 5%CO_2_). Absorbance was measured at a wavelength of 450 nm using a microplate reader. Cell viability was calculated using the following formula: Cell viability (%) = [(As − Ab)/(Ac − Ab)] × 100, where As, Ac, and Ab represent the absorbance values of the sample (drug-treated), control (untreated), and blank (medium-only) wells, respectively.

### 2.6. Lactate Dehydrogenase (LDH) Release Assay

Cytotoxicity was assessed by measuring the release of LDH into the culture medium using a commercial LDH assay kit (Beyotime, Shanghai, China, Cat# C0016). Following drug treatments, the assay was performed according to the manufacturer’s instructions with a minor modification. Briefly, 1 h prior to the designated measurement time point, the LDH release reagent was added to the designated “maximum LDH activity control” wells and mixed gently. The plate was then returned to the incubator. At the measurement time point, the plate was centrifuged at 400× *g* for 5 min to pellet cell debris. Subsequently, 120 µL of supernatant from each well was transferred to a new plate, mixed with 60 µL of LDH assay working solution, and incubated at room temperature, protected from light, for 30 min. The absorbance of the supernatant was measured at 490 nm using a microplate reader. The percentage of LDH release was calculated as follows: LDH release (%) = [(AT − AC)/(AM − AC)] × 100, where AT = Absorbance of drug-treated sample wells, AC = Absorbance of untreated control wells (spontaneous LDH release), and AM = Absorbance of maximum LDH activity control wells (total releasable LDH).

### 2.7. Transmission Electron Microscopy (TEM)

Following the removal of culture medium, cells were fixed with a sufficient volume of ice-cold 2.5% glutaraldehyde at 4 °C for 1 h. After fixation, cells were gently scraped, pelleted by centrifugation, and processed through a standard protocol of dehydration and resin embedding. Ultrathin sections (approximately 60 nm in thickness) were prepared using an ultramicrotome. Cellular ultrastructure, with a specific focus on mitochondrial morphology and the presence of autophagic vacuoles (mitophagy) and ferroptotic features, was examined using a Tecnai G2 Spirit BioTWIN transmission electron microscope (FEI, Hillsboro, OR, USA).

### 2.8. Detection of Intracellular Fe^2+^

Intracellular Fe^2+^ levels were assessed using the fluorescent probe FerroOrange (Dojindo, Kumamoto, Japan, Cat# F374). Cells were seeded on glass-bottom confocal dishes and subjected to the specified drug treatments. After treatment, cells were washed once with PBS and incubated with 1 μmol/L FerroOrange working solution for 30 min under standard culture conditions. Fluorescence imaging was performed using a laser scanning confocal microscope.

### 2.9. Detection of Lipid Peroxidation

Intracellular lipid peroxidation was assessed using the Lipid Peroxidation Probe-BDP 581/591 C11 (Cat# L267, Dojindo, Kumamoto, Japan). Cells were seeded on glass-bottom confocal dishes and subjected to the specified treatments. After treatment, cells were washed once with PBS and incubated with the BDP 581/591 C11 working solution for 30 min at 37 °C. Fluorescence images were acquired using a laser scanning confocal microscope. The extent of lipid peroxidation was quantified as the ratio of green fluorescence intensity (oxidized probe) to red fluorescence intensity (reduced probe).

### 2.10. Measurement of Mitochondrial Membrane Potential (MMP)

Mitochondrial membrane potential was measured using the JC-1 Assay Kit (Beyotime, Shanghai, China, Cat# C2006). Cells cultured in a 6-well plate were washed once with PBS after removal of the culture medium. Subsequently, 1 mL of fresh culture medium was mixed with 1 mL of JC-1 staining working solution and added to each well. Cells were incubated with the dye at 37 °C for 20 min. Following incubation, the staining solution was aspirated, and cells were washed twice with the provided 1X JC-1 staining buffer. Finally, 2 mL of fresh culture medium was added to each well. Fluorescence images were captured using a fluorescence microscope. The MMP was quantified by calculating the ratio of green (monomers, low MMP) to red (aggregates, high MMP) fluorescence intensity.

### 2.11. Quantitative Real-Time PCR (qRT-PCR)

Total RNA was isolated from MCs using TRIzol reagent (Vazyme, Nanjing, China). RNA concentration and purity (A260/A280 ratio) were determined using a NanoDrop™ spectrophotometer (Thermo Fisher Scientific, Waltham, MA, USA). According to the manufacturer’s protocol, 1 µg of total RNA was reverse-transcribed into complementary DNA (cDNA) using the HiScript III RT SuperMix for qPCR (Cat# R323-01, Vazyme, Nanjing, China). Quantitative PCR (qPCR) was then performed with ChamQ Universal SYBR qPCR Master Mix (Vazyme, Nanjing, China) on a real-time PCR detection system. The mRNA expression levels of target genes were normalized to that of β-actin, which served as an internal control. Relative gene expression was calculated using the comparative 2^−ΔΔCt^ method. All primer sequences are provided in Supplementary Materials (Table S2).

### 2.12. Western Blot Analysis

#### 2.12.1. Total Protein Extraction

Total cellular protein was extracted using RIPA lysis buffer (Servicebio, Wuhan, China, Cat# G2002). Following drug treatment, cells were washed once with PBS and lysed on ice for 10 min with RIPA buffer supplemented with 1 mM phenylmethylsulfonyl fluoride (PMSF). Cell lysates were then sonicated on ice and clarified by centrifugation at 10,000× *g* for 10 min at 4 °C. The resulting supernatant was collected as the total protein lysate.

#### 2.12.2. Subcellular Fractionation

Cytosolic and mitochondrial proteins were fractionated using a Mitochondrial Isolation Kit (Beyotime, Shanghai, China, Cat# C3601). Briefly, after drug treatment, cells were trypsinized, harvested by centrifugation at 100× *g* for 10 min, and washed with ice-cold PBS. The cell pellet was resuspended in the supplied mitochondrial isolation reagent containing 1 mM PMSF and incubated on ice for 10 min. Cells were then homogenized mechanically until >50% cell breakage was achieved, as assessed by trypan blue exclusion. The homogenate was centrifuged at 600× *g* for 5 min at 4 °C to remove nuclei and unbroken cells. The supernatant was further centrifuged at 11,000× *g* for 10 min at 4 °C to pellet the mitochondrial fraction. The resulting supernatant (post-mitochondrial fraction) was centrifuged at 12,000× *g* for 10 min at 4 °C to obtain the final cytosolic protein fraction. The mitochondrial pellet was lysed in mitochondrial lysis buffer containing 1 mM PMSF.

#### 2.12.3. Immunoblotting

Protein concentrations were determined using a BCA assay. Equal amounts of protein were separated by SDS-PAGE and transferred to polyvinylidene difluoride (PVDF) membranes (Cat. #E802, Vazyme, Nanjing, China). After blocking, membranes were incubated overnight at 4 °C with primary antibodies (listed in Supplementary Materials [Table S1]), followed by incubation with appropriate HRP-conjugated secondary antibodies. Protein bands were visualized using enhanced chemiluminescence (ECL, Cat. #MA0187, Meilunbio, Dalian, China) substrate and imaged.

### 2.13. Immunofluorescence

Cells were cultured on glass coverslips and fixed with 4% paraformaldehyde at room temperature for 15 min. After fixation, cells were permeabilized and blocked with 0.3% Triton X-100 in donkey serum for 30 min. Coverslips were then incubated overnight at 4 °C with primary antibodies (see Supplementary Materials [Table S1]). After washing, cells were incubated with fluorescently labeled secondary antibodies (ANT024s, Antgene, Wuhan, China) for 1 h at 37 °C in the dark. Nuclei were counterstained with DAPI (Cat. #ANT063, Antgene, Wuhan, China). Images were acquired using a Nikon A1R SI confocal laser scanning microscope (Nikon, Tokyo, Japan).

### 2.14. Lentiviral Transduction for PINK1 Overexpression

To achieve PINK1 overexpression, primary MCs were transduced with a lentiviral vector carrying rat Pink1 (GV492, GeneChem, Shanghai, China). The vector contained a Ubc promoter–driven Pink1 expression cassette with a 3FLAG tag and a CBh promoter–driven gcGFP–IRES–puromycin cassette. For transduction, the standard culture medium was replaced 12 h post-seeding with fresh medium containing the lentiviral particles at a viral titer of 1 × 1011 vg/mL. The viral supernatant was removed after a 72 h incubation period and replaced with standard growth medium. Cells were then cultured for an additional 48 h to allow for transgene expression prior to subsequent experiments.

### 2.15. Transcriptome Sequencing and Data Analysis

Total RNA was extracted from primary rat cochlear MCs using VeZol reagent (Vazyme, Nanjing, China). RNA quality was assessed using a NanoDrop 2000 spectrophotometer (Thermo Fisher Scientific, Waltham, MA, USA) and an Agilent 5300 Fragment Analyzer (Agilent Technologies, Santa Clara, CA, USA). Library preparation and paired-end 150 bp sequencing were performed by Shanghai Majorbio Bio-Pharm Technology Co., Ltd. (Shanghai, China).

Raw reads were quality-filtered using fastp and aligned to the rat reference genome Rnor_6.0 using HISAT2 v2.2.1. Reference-guided transcript assembly was performed using StringTie v2.2.1, and gene abundance was quantified using RSEM v1.3.3 and expressed as transcripts per million (TPM). Differentially expressed genes were identified using DESeq2 v1.38.3, with FDR <0.05 and |log_2_FC| ≥1 as the significance criteria. Gene Ontology (GO) enrichment analyses and gene set enrichment analysis (GSEA) were conducted using the Majorbio Cloud Platform. The RNA-sequencing data generated in this study have been deposited in the NCBI Sequence Read Archive (SRA) under BioProject accession number PRJNA1402639.

### 2.16. Statistical Analysis

Data are presented as the mean ± SD. Statistical analyses were performed using GraphPad Prism version 8.0 (GraphPad Software, San Diego, CA, USA). Data distributions and homogeneity of variances were examined before analysis. Comparisons between two independent groups were performed using an unpaired two-tailed Student’s *t*-test. Comparisons among three or more groups involving a single independent variable were performed using ordinary one-way ANOVA followed by Tukey’s multiple-comparisons test. ABR threshold data were analyzed using two-way repeated-measures ANOVA, with the Greenhouse–Geisser correction, followed by Tukey’s multiple-comparisons test. A two-sided *p* value < 0.05 was considered statistically significant. Statistical significance is indicated as follows: ns, not significant; * *p* < 0.05; ** *p* < 0.01; *** *p* < 0.001; and **** *p* < 0.0001.

## 3. Results

### 3.1. Cisplatin Induces Ferroptosis in MCs

While cisplatin has been shown to induce apoptosis, pyroptosis, and necrosis in MCs [7,9], its ability to trigger ferroptosis remains unclear. We therefore treated cultured primary rat cochlear MCs with cisplatin to investigate whether ferroptosis contributes to cisplatin-induced MC injury.

**Figure 1:**
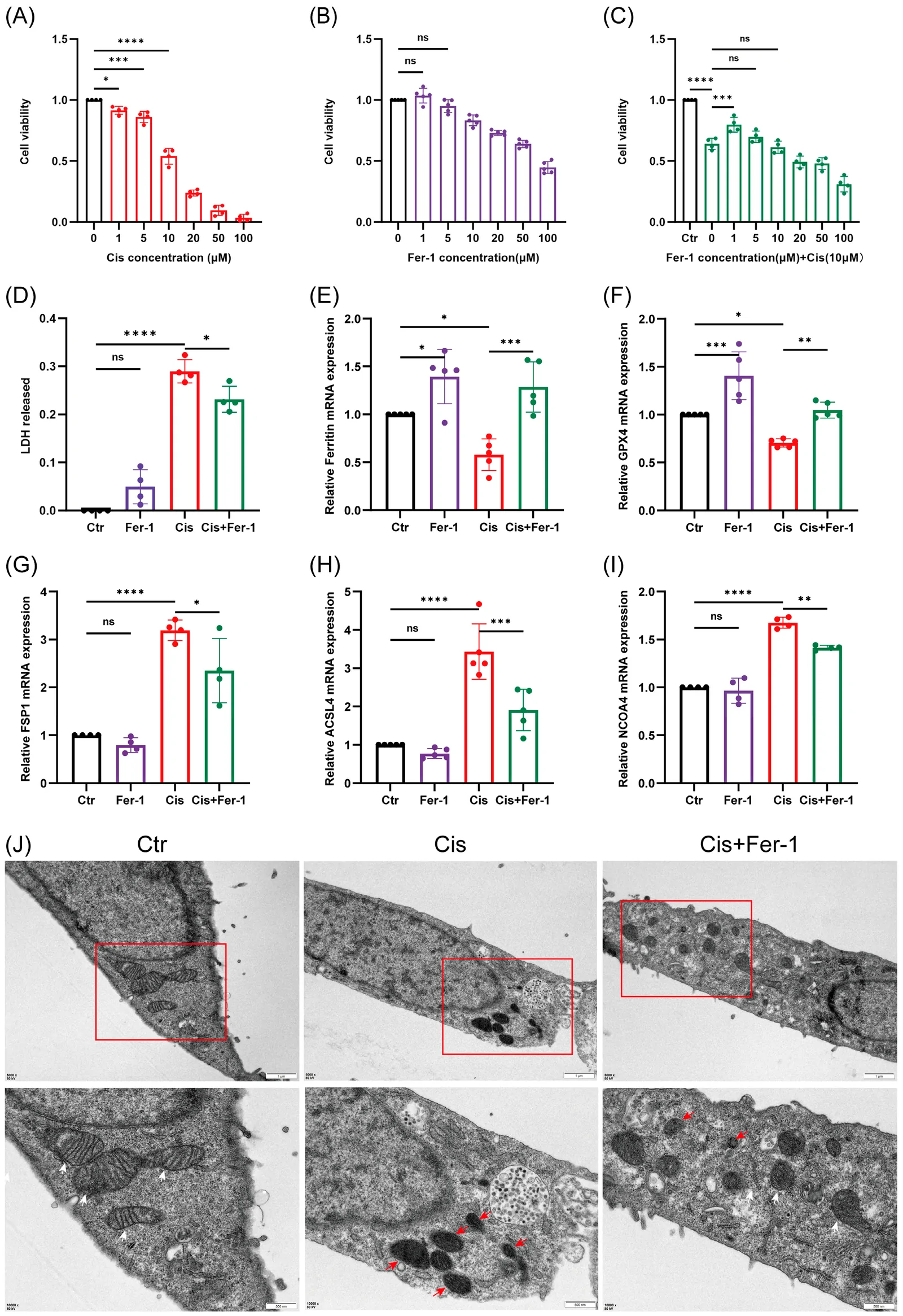
Cisplatin induces ferroptosis in cultured primary rat cochlear marginal cells (MCs), which is attenuated by Ferrostatin-1. (**A**–**C**) Cell viability of MCs treated with cisplatin alone (**A**) or Fer-1 alone (**B**), or pretreated with Fer-1 followed by cisplatin exposure (**C**), as assessed using the CCK-8 assay. (**D**) LDH release assay. (**E**–**I**) Relative mRNA expression levels of ferroptosis-related genes: Ferritin (**E**), GPX4 (**F**), FSP1 (**G**), ACSL4 (**H**), and NCOA4 (**I**), as determined by qPCR analysis. (**J**) Representative transmission electron microscopy (TEM) images of MC mitochondria. White arrowheads, normal mitochondria; red arrowheads, mitochondria with ferroptotic morphological alterations (condensed, reduced cristae). Scale bars: 1 µm (upper panel), 500 nm (lower panel). Data are mean ± SD (*n* = 4–5). ns, not significant; * *p* < 0.05; ** *p* < 0.01; *** *p* < 0.001; **** *p* < 0.0001. Cis, cisplatin; Fer-1, Ferrostatin-1. ACSL4: acyl-CoA synthetase long-chain family member 4; GPX4: glutathione peroxidase 4; FSP1: ferroptosis suppressor protein 1; NCOA4: nuclear receptor coactivator 4.

**Figure 2:**
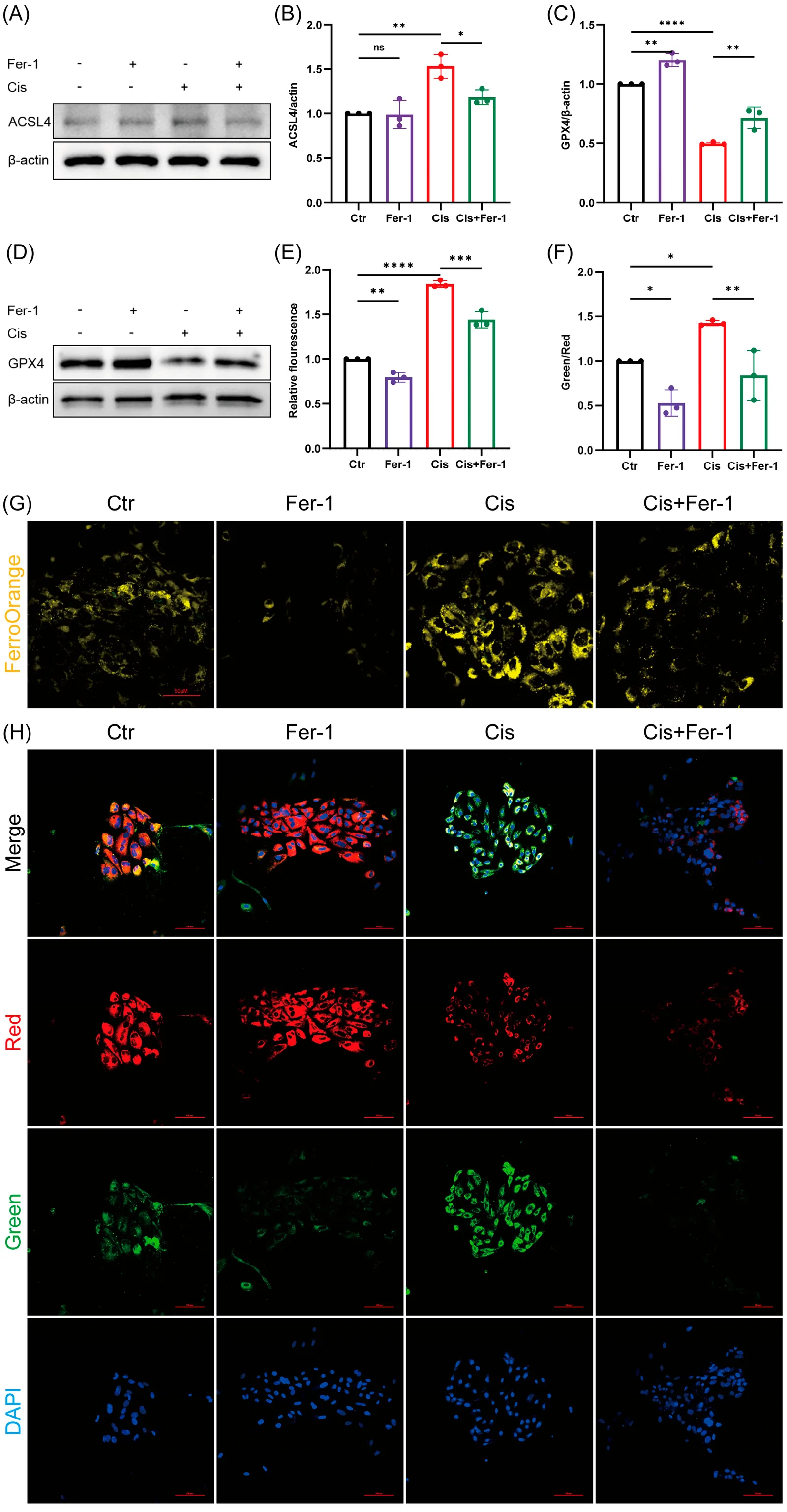
Fer-1 attenuates cisplatin-induced lipid peroxidation and iron overload in MCs. (**A**–**D**) Western blot analysis of key ferroptosis-related proteins: ACSL4 (**A**,**B**) and GPX4 (**C**,**D**). (**E**,**G**) Intracellular labile Fe^2+^ levels assessed by FerroOrange fluorescence: representative images (**G**); quantification (**E**). Scale bar: 50 µm. (**F**,**H**) Lipid peroxidation assessed by BDP 581/591 C11 probe fluorescence: representative images (**H**); quantification of the green-to-red fluorescence intensity ratio (**F**). Scale bar: 100 µm. Data are mean ± SD (*n* = 3). ns, not significant; * *p* < 0.05; ** *p* < 0.01; *** *p* < 0.001; **** *p* < 0.0001.

Cisplatin treatment significantly reduced cell viability, as determined by the CCK-8 assay, and increased cytotoxicity, as measured by LDH release (Figure 1A,D). At the molecular level, cisplatin markedly downregulated the expression of key ferroptosis suppressors GPX4, while upregulating the ferroptosis promoter ACSL4, indicative of elevated lipid peroxidation (Figure 1F–H and Figure 2A–D). Concomitantly, increased nuclear receptor coactivator 4 (NCOA4) expression together with reduced ferritin expression was consistent with altered iron-storage regulation and a possible increase in ferritinophagic activity. FSP1 mRNA expression was also increased (Figure 1G). TEM revealed hallmark mitochondrial alterations of ferroptosis in cisplatin-treated MCs, including increased membrane density, condensation, and loss of cristae (Figure 1J; white arrows, normal mitochondria; red arrows, ferroptotic mitochondria). Consistent with these findings, measurements showed increased intracellular labile Fe^2+^ levels and lipid peroxidation. The FerroOrange probe showed a pronounced increase in intracellular Fe^2+^ fluorescence, supporting increased intracellular iron accumulation (Figure 2E,G). Similarly, using the BDP 581/591 C11 probe, which shifts its fluorescence from red to green upon oxidation, we observed a significantly higher green-to-red fluorescence ratio in cisplatin-treated cells, demonstrating robust lipid peroxidation (Figure 2F,H).

Crucially, these cisplatin-induced phenotypes were substantially rescued by the specific ferroptosis inhibitor Fer-1. Fer-1 pretreatment attenuated cell death, lipid peroxidation, and iron overload, ameliorated mitochondrial abnormalities, and normalized the expression of ferroptosis-related markers (Figure 1C–J and Figure 2A–H).

In summary, multi-faceted evidence from cell death assays, biochemical measurements of ferroptotic hallmarks (iron overload and lipid peroxidation), ultrastructural analysis, and molecular profiling collectively support the involvement of ferroptosis in cisplatin-induced MC injury.

### 3.2. Cisplatin Impairs Mitochondrial Function and Inhibits Mitophagy

**Figure 3:**
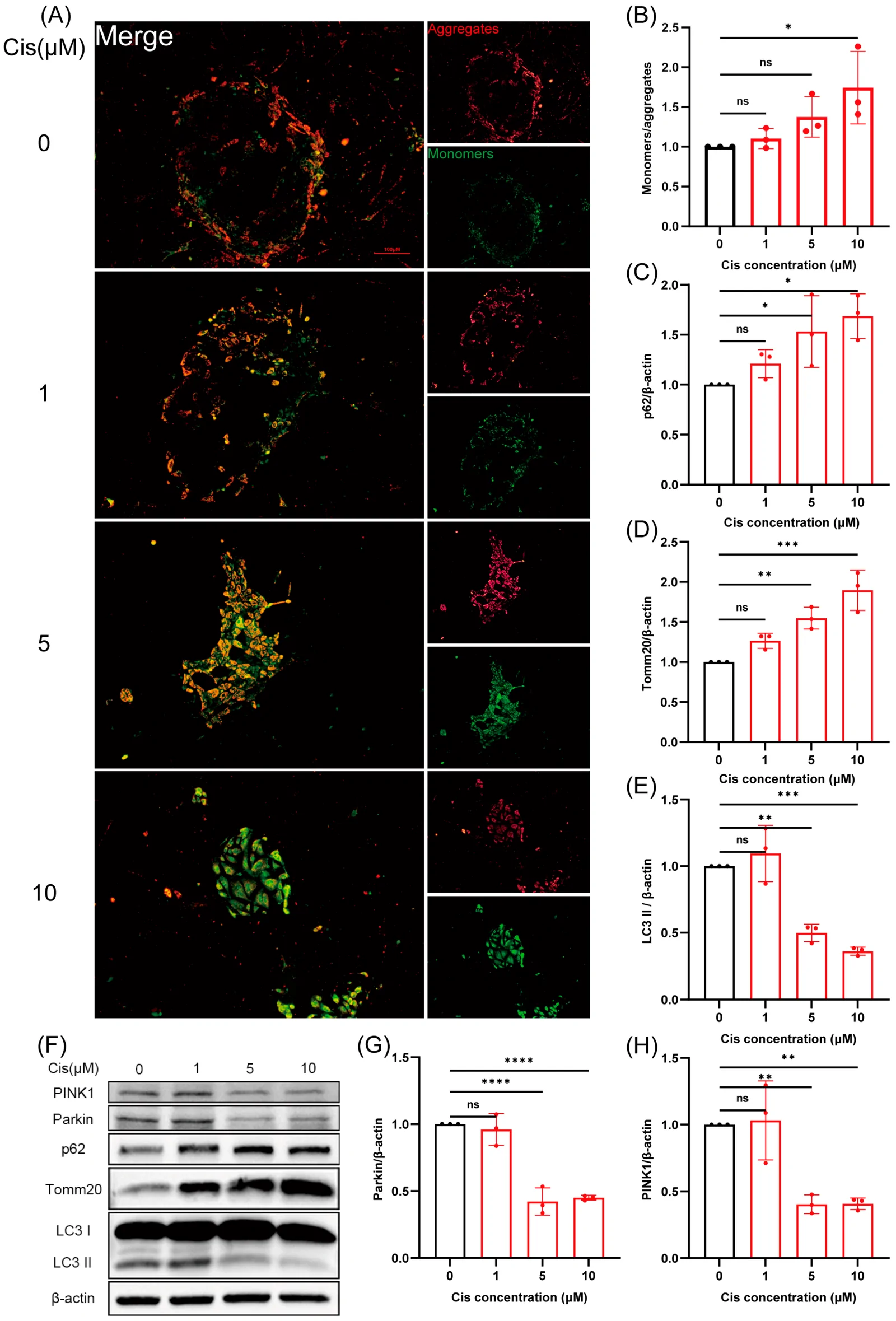
Cisplatin induces mitochondrial dysfunction and suppresses mitophagy in MCs. (**A**,**B**) Mitochondrial membrane potential assessed by JC-1 probe fluorescence: representative images (**A**) (red, JC-1 aggregates; green, JC-1 monomers) and quantification of the monomer-to-aggregate fluorescence intensity ratio (**B**). Scale bar: 100 µm. (**C**–**H**) Western blot analysis (**F**) and quantification of key proteins involved in mitophagy regulation: p62 (**C**), Tomm20 (**D**), LC3 II (**E**), Parkin (**G**), and PINK1 (**H**). Data are presented as mean ± SD (*n* = 3). Statistical significance: ns, not significant; * *p* < 0.05; ** *p* < 0.01; *** *p* < 0.001; **** *p* < 0.0001. PINK1: PTEN-induced putative kinase 1.

Assessment of MMP using the JC-1 probe revealed a pronounced shift from red (high MMP in controls) to green fluorescence (low MMP) in cisplatin-treated MCs, indicating mitochondrial depolarization and functional impairment (Figure 3A,B).

**Figure 4:**
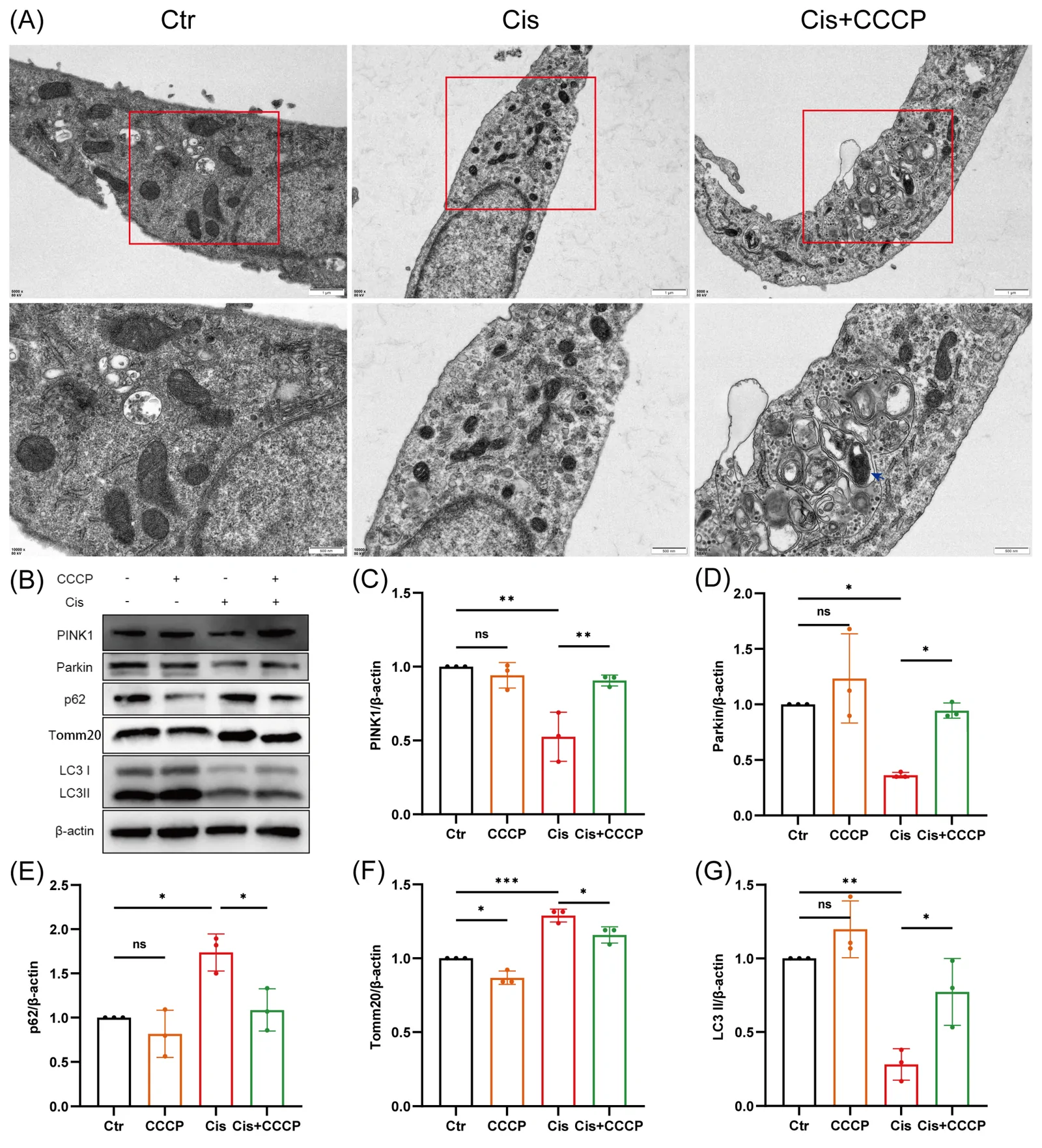
The mitophagy inducer carbonyl cyanide m-chlorophenyl hydrazone (CCCP) rescues cisplatin-impaired mitophagy in MCs. (**A**) Representative TEM images of mitochondrial ultrastructure. Blue arrowheads indicate mitophagosomes. Scale bars: 1 µm (upper panel), 500 nm (lower panel). (**B**–**G**) Western blot analysis (**B**) and quantification of key mitophagy-associated proteins: PINK1 (**C**), Parkin (**D**), p62 (**E**), Tomm20 (**F**), and LC3 II (**G**). Data are presented as mean ± SD (*n* = 3). Statistical significance: ns, not significant; * *p* < 0.05; ** *p* < 0.01; *** *p* < 0.001.

**Figure 5:**
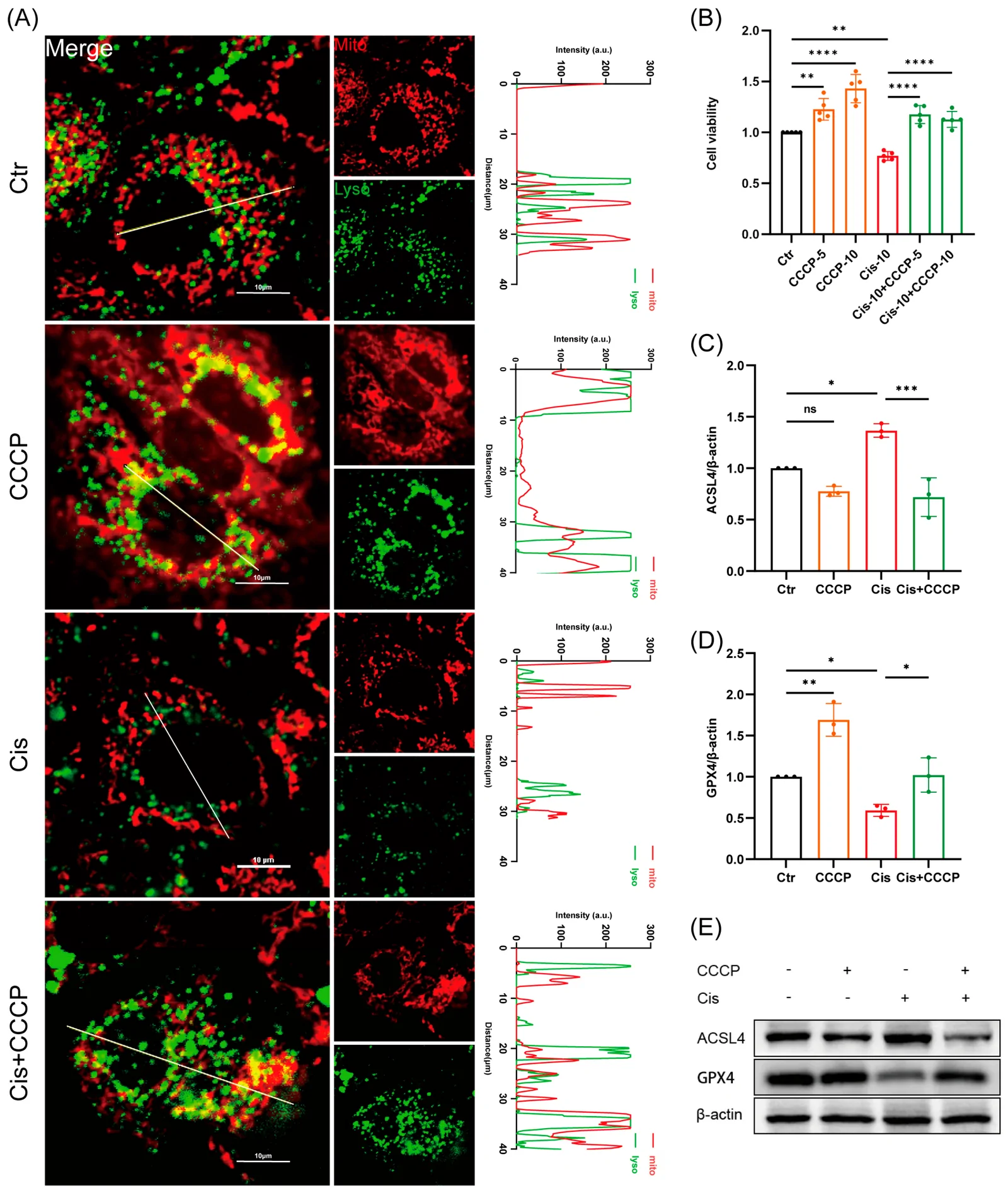
Restoration of mitophagy attenuates cisplatin-induced ferroptosis in MCs. (**A**) Representative images show colocalization of MitoTracker (red, mitochondria) and LysoTracker (green, lysosomes) and colocalization analysis. Scale bar: 10 µm. (**B**) Cell viability was assessed by CCK-8 assay. (**C**–**E**) Western blot analysis (**E**) and quantification of key ferroptosis regulators: ACSL4 (**C**) and GPX4 (**D**). Data are presented as mean ± SD (*n* = 3–5). Statistical significance: ns, not significant; * *p* < 0.05; ** *p* < 0.01; *** *p* < 0.001; **** *p* < 0.0001.

Western blot analysis of key mitophagy markers showed that cisplatin treatment downregulated the autophagosome-associated protein LC3 II and led to the accumulation of the autophagy receptor p62, suggesting impaired autophagic activity. Concurrently, the mitochondrial outer membrane protein Tomm20 increased, and the PINK1-Parkin signaling axis was suppressed, further supporting impaired mitophagic activity (Figure 3C–H). Consistent with these molecular changes, TEM revealed a striking accumulation of structurally abnormal mitochondria in cisplatin-treated cells. Notably, characteristic mitophagic structures, such as mitophagosomes and autolysosomes, were absent (Figure 4A). To assess mitochondrial delivery to lysosomes as an indicator of mitophagic activity, we examined the colocalization of MitoTracker-labeled mitochondria with LysoTracker-labeled lysosomes. Cisplatin treatment reduced the level of mitochondria-lysosome colocalization, indicating impaired delivery of mitochondria to lysosomes for degradation (Figure 5A).

In summary, cisplatin disrupts MMP and inhibits the PINK1-Parkin-associated mitophagic pathway. This leads to a failure in clearing damaged mitochondria, resulting in their pathological accumulation.

### 3.3. Induction of Mitophagy Attenuates Cisplatin-Induced Ferroptosis

Given that cisplatin concurrently inhibits mitophagy and induces ferroptosis in MCs, we next investigated their functional interplay. To this end, we employed the mitochondrial uncoupler and mitophagy inducer CCCP to restore mitophagic activity and assess its impact on cisplatin-induced ferroptosis.

Notably, TEM showed that co-treatment with CCCP substantially enhanced mitophagic activity in cisplatin-exposed MCs. This was characterized by the appearance of mitophagosomes (blue arrowheads) and a concomitant decrease in the accumulation of structurally abnormal mitochondria, compared to cisplatin treatment alone (Figure 4A). Correspondingly, Western blot analysis confirmed that CCCP co-treatment effectively reversed the cisplatin-induced alterations in key mitophagy markers: it elevated LC3 II levels, reduced p62 and Tomm20 accumulation, and restored PINK1 and Parkin expression (Figure 4B–G). Supporting these observations, confocal microscopy analysis revealed that CCCP co-treatment significantly restored mitochondria–lysosome colocalization, indicating a recovery of functional mitophagic flux impaired by cisplatin (Figure 5A). Taken together, these results suggest that pharmacological induction of mitophagy by CCCP effectively counteracts cisplatin-induced mitophagy suppression in MCs.

To assess the functional consequence of restored mitophagy on ferroptosis, we analyzed the expression of key ferroptotic markers. Co-treatment with CCCP significantly reversed the cisplatin-induced downregulation of the ferroptosis inhibitor GPX4 and upregulation of the ferroptosis promoter ACSL4 at the protein level (Figure 5C–E). Consistent with this protective molecular profile, cell viability was significantly higher in the Cis+CCCP group than in the cisplatin-only group, as measured by the CCK-8 assay (Figure 5B).

In summary, restoring mitophagic flux effectively mitigates cisplatin-induced ferroptosis in MCs, as evidenced by the reversal of key ferroptotic marker expression and reduced cell death. This supports a protective, inhibitory role for mitophagy in the regulation of cisplatin-induced ferroptosis.

### 3.4. PINK1 Overexpression Attenuates Cisplatin-Induced Mitophagy Impairment and Ferroptosis

Given that the PINK1-Parkin pathway is the canonical regulator of mitophagy and that CCCP induces mitophagy via this axis, we next investigated whether cisplatin triggers ferroptosis by specifically suppressing PINK1-associated mitophagy.

Western blot analysis confirmed that cisplatin treatment significantly downregulated both PINK1 and Parkin proteins in MCs. This suppression was substantially reversed by co-treatment with CCCP, which restored PINK1 and Parkin expression (Figure 3F–H and Figure 4B–D). These data imply that cisplatin inhibits mitophagy, at least in part, by impairing the PINK1-Parkin pathway, and that CCCP rescues this pathway. Together with our finding that CCCP alleviates cisplatin-induced ferroptosis by restoring mitophagy, this pointed to a pivotal role for PINK1-associated mitophagy in regulating ferroptosis.

**Figure 6:**
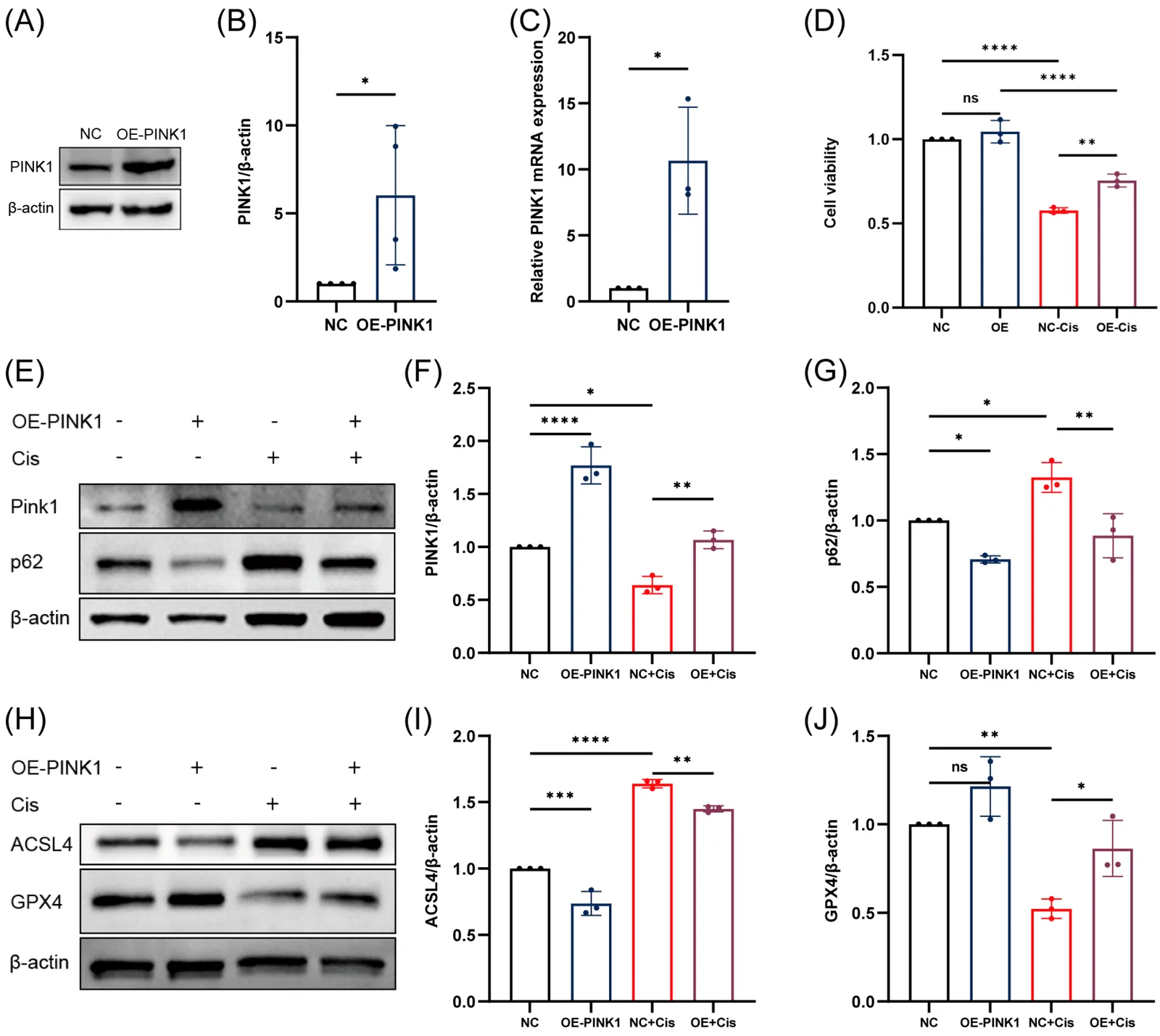
PINK1 overexpression restores mitophagy and attenuates cisplatin-induced ferroptosis in MCs. (**A**–**C**). Validation of PINK1 overexpression. Western blot analysis of PINK1 protein (**A**,**B**) and qPCR analysis of PINK1 mRNA (**C**) in MCs transduced with a PINK1-overexpressing (OE-PINK1) or control vector. (**D**) Cell viability assessed by CCK-8 assay in the indicated treatment groups. (**E**–**G**) Western blot analysis (**E**) and quantification of PINK1 (**F**) and p62 (**G**). (**H**–**J**) Western blot analysis (**H**) and quantification of ACSL4 (**I**) and GPX4 (**J**). Data are presented as mean ± SD (*n* = 3–4). Statistical significance: ns, not significant; * *p* < 0.05; ** *p* < 0.01; *** *p* < 0.001; **** *p* < 0.0001.

To directly test this hypothesis, we overexpressed PINK1 (OE-PINK1) in MCs, confirming efficiency via Western blot and qPCR (Figure 6A–C). Similar to CCCP, PINK1 overexpression significantly rescued mitophagy in cisplatin-treated cells, as indicated by reduced p62 levels (Figure 6G). This was accompanied by a marked attenuation of ferroptotic signatures, including decreased ACSL4 and increased GPX4 expression (Figure 6H–J). Consequently, cell viability was significantly improved in OE-PINK1+Cis cells compared to NC+Cis (Figure 6D). Collectively, these findings support the involvement of PINK1-associated mitophagy in the regulation of cisplatin-induced ferroptosis in MCs. Cisplatin promotes ferroptosis by inhibiting the PINK1-Parkin pathway and blocking mitophagic clearance of damaged mitochondria, whereas activation of PINK1 restores mitophagy and exerts a protective effect against cisplatin-induced ferroptosis.

### 3.5. Induction of Mitophagy Attenuates Cisplatin-Induced BLB Damage and Hearing Loss In Vivo

DFO, a canonical iron chelator, directly inhibits ferroptosis by reducing the intracellular labile iron pool. Intriguingly, DFO has also been identified as a potent mitophagy inducer [33]. Prior work has shown that intratympanic DFO at 800 μg/mL alleviates otitis and hearing loss [34], and intraperitoneal injection of another iron chelator, deferiprone (DFP, 100 mg/kg), reduced neomycin-induced apoptosis of HCs by clearing damaged mitochondria [35]. Therefore, we evaluated whether DFO could protect against cisplatin ototoxicity in vivo by modulating the mitophagy-ferroptosis axis.

**Figure 7:**
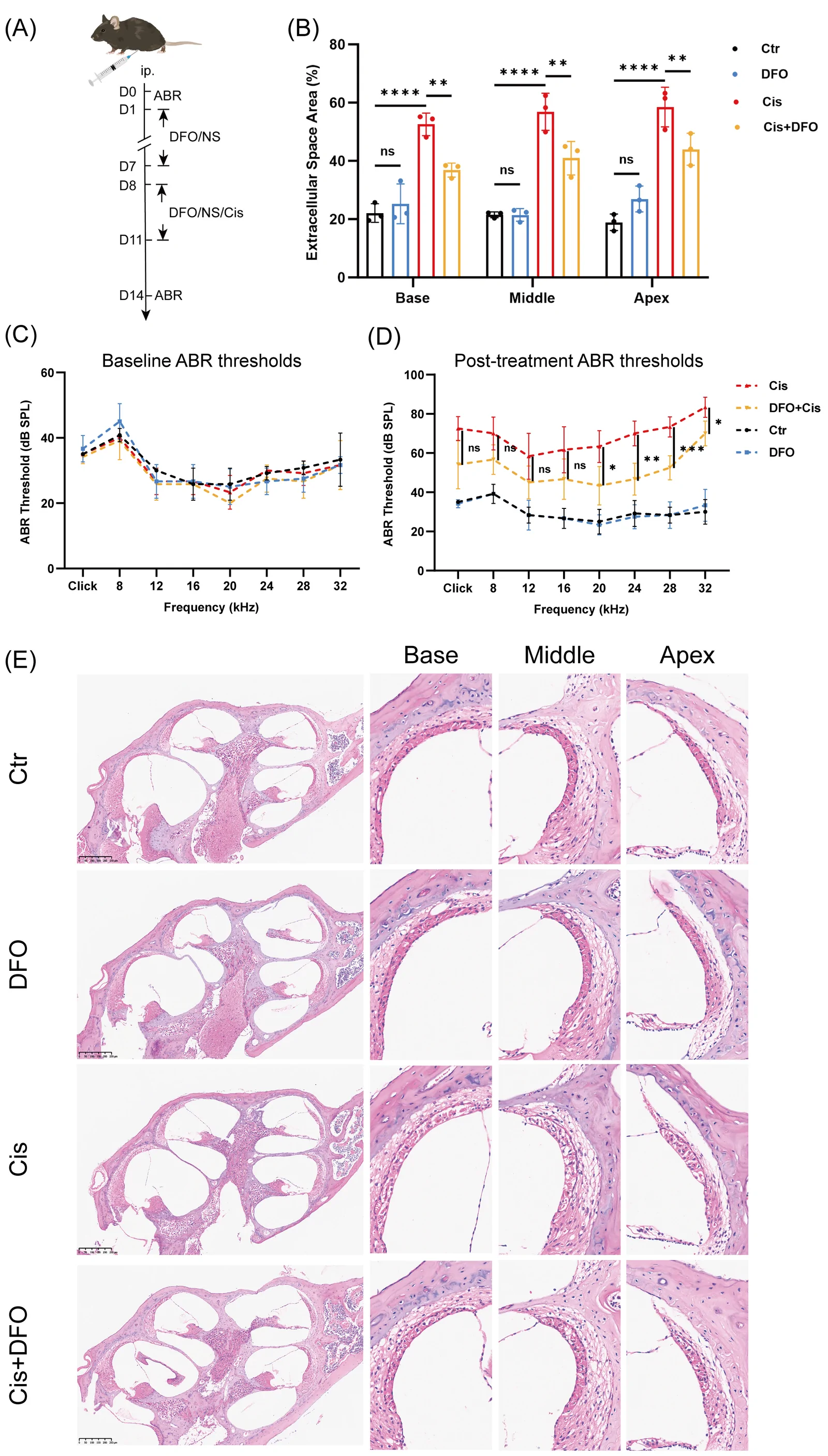
Deferoxamine (DFO) attenuates cisplatin-induced hearing loss and BLB disruption in mice. (**A**) Schematic illustration of the in vivo treatment protocol. (**B**,**E**). Representative hematoxylin and eosin (H&E)-stained cross-sections of the cochlea (**E**), focusing on the stria vascularis and quantitative analysis of the ratio of strial vascularis intercellular space area to total strial vascularis cross-sectional area (**B**). Scale bar = 250 μm. (**C**,**D**) Assessment of auditory function by auditory brainstem response (ABR). Baseline hearing thresholds before treatment (**C**) and post-treatment hearing thresholds (**D**) across frequencies are shown. Data are presented as mean ± SD (*n* = 3–6). ns, not significant; * *p* < 0.05; ** *p* < 0.01; *** *p* < 0.001; **** *p* < 0.0001.

**Figure 8:**
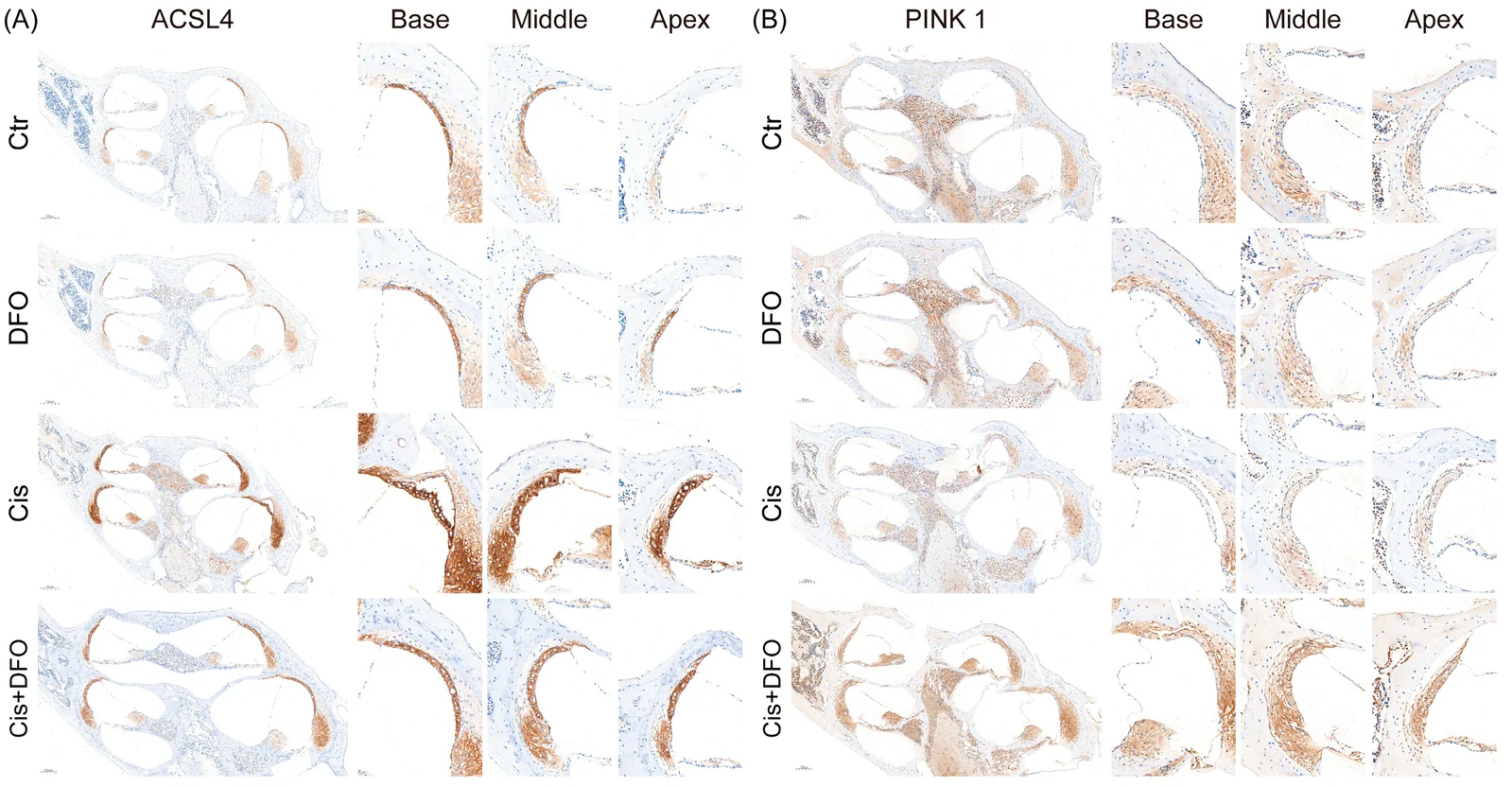
DFO promotes mitophagy and inhibits ferroptosis in the cochlear stria vascularis in vivo. (**A**,**B**) Representative immunohistochemical staining of cochlear sections showing the expression of the ferroptosis driver ACSL4 (**A**) and the mitophagy initiator PINK1 (**B**) in the lateral wall. Scale bar: 100 µm.

ABR measurements showed that intraperitoneal cisplatin administration significantly elevated hearing thresholds across all tested frequencies, with more pronounced impairment at high frequencies (28–32 kHz). Notably, DFO treatment markedly reduced high-frequency hearing thresholds compared with the cisplatin-only group, indicating that DFO effectively attenuated cisplatin-induced high-frequency hearing loss (Figure 7C,D). H&E staining of cochlear sections revealed pronounced pathological alterations in the SV of cisplatin-treated mice, characterized by tissue edema, widened extracellular spaces, and disruption of the BLB structure. In contrast, DFO intervention significantly alleviated these pathological changes in the SV (Figure 7B,E). Furthermore, immunohistochemical analysis of cochlear tissues showed that, compared with the cisplatin-only group, the DFO+Cis group exhibited stronger PINK1 immunoreactivity and weaker ACSL4 immunoreactivity (Figure 8A,B).

Collectively, these in vivo findings demonstrate that DFO attenuated cisplatin-induced BLB damage and high-frequency hearing loss, accompanied by increased PINK1 expression and decreased ACSL4 expression in the cochlear SV. These changes were consistent with the molecular pattern observed in vitro.

### 3.6. Mitophagy Induction Is Associated with Altered ACSL4 Subcellular Distribution

During mitophagic clearance, mitochondrial components and associated proteins can be removed together with damaged mitochondria. The subcellular localization of ferroptosis regulators, including ACSL4 and GPX4, is dynamically regulated, and their redistribution between the cytosol and mitochondria may influence ferroptotic responses. Previous work showed that mitophagy attenuated ferroptosis by inhibiting ACSL4 mitochondrial translocation [31]. In contrast, we observed reduced mitochondrial localization of ACSL4 under cisplatin-induced mitophagy impairment, despite the concomitant enhancement of lipid peroxidation and ferroptosis. This apparently divergent observation prompted us to investigate how mitophagy affects the subcellular distribution of ACSL4 in cochlear MCs.

**Figure 9:**
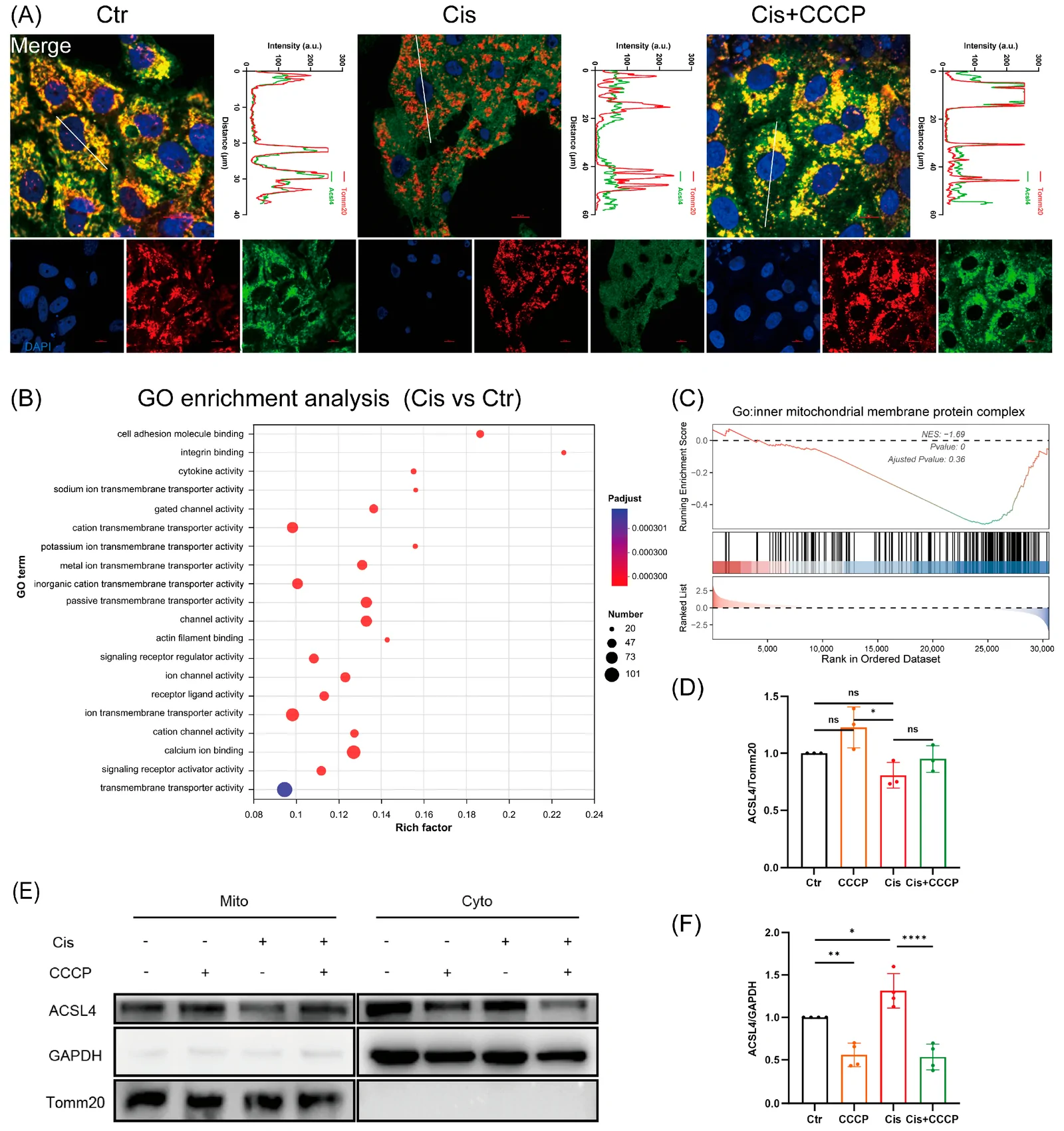
Cisplatin alters the subcellular distribution of ACSL4 in MCs. (**A**) Representative immunofluorescence images showing the co-localization of ACSL4 (green) and Tomm20 (red) in MCs. Scale bar: 10 µm. (**B**) GO enrichment analysis of differentially expressed genes between control and cisplatin-treated MCs. (**C**) GSEA plot showing a negative enrichment score of the mitochondrial membrane protein complex pathway upon cisplatin treatment. (**D**–**F**) Western blot analysis (**E**) and quantification of ACSL4 protein levels in isolated mitochondrial (**D**) and cytosolic (**F**) fractions. Data are presented as mean ± SD (*n* = 3). Statistical significance: ns, not significant; * *p* < 0.05; ** *p* < 0.01; **** *p* < 0.0001.

GO enrichment analysis of the RNA-seq data showed that cisplatin significantly affected pathways related to molecular functions related to ion transmembrane transport, channel activity, and receptor binding (Figure 9B). GSEA further revealed downregulation of pathways associated with mitochondrial membrane protein complexes (Figure 9C), suggesting disruption of mitochondrial membrane-associated processes. Immunofluorescence analysis demonstrated substantial co-localization of ACSL4 with the mitochondrial marker Tomm20 in control MCs. Following cisplatin treatment, ACSL4 exhibited a more diffuse cytosolic distribution, accompanied by a marked reduction in its co-localization with Tomm20. Co-treatment with CCCP partially restored ACSL4–Tomm20 co-localization (Figure 9A). Consistent with these observations, subcellular fractionation followed by Western blotting showed that CCCP co-treatment significantly reduced cytosolic ACSL4 levels compared with cisplatin treatment alone, whereas no corresponding accumulation of ACSL4 was detected in the mitochondrial fraction (Figure 9D–F). These findings are consistent with a model in which CCCP facilitates the mitochondrial targeting of cytosolic ACSL4 while simultaneously promoting mitophagic clearance, thereby reducing the cytosolic ACSL4 pool without causing its detectable accumulation in the mitochondrial fraction. However, direct tracking of ACSL4 turnover and lysosomal degradation will be required to validate this proposed mechanism.

**Figure 10:**
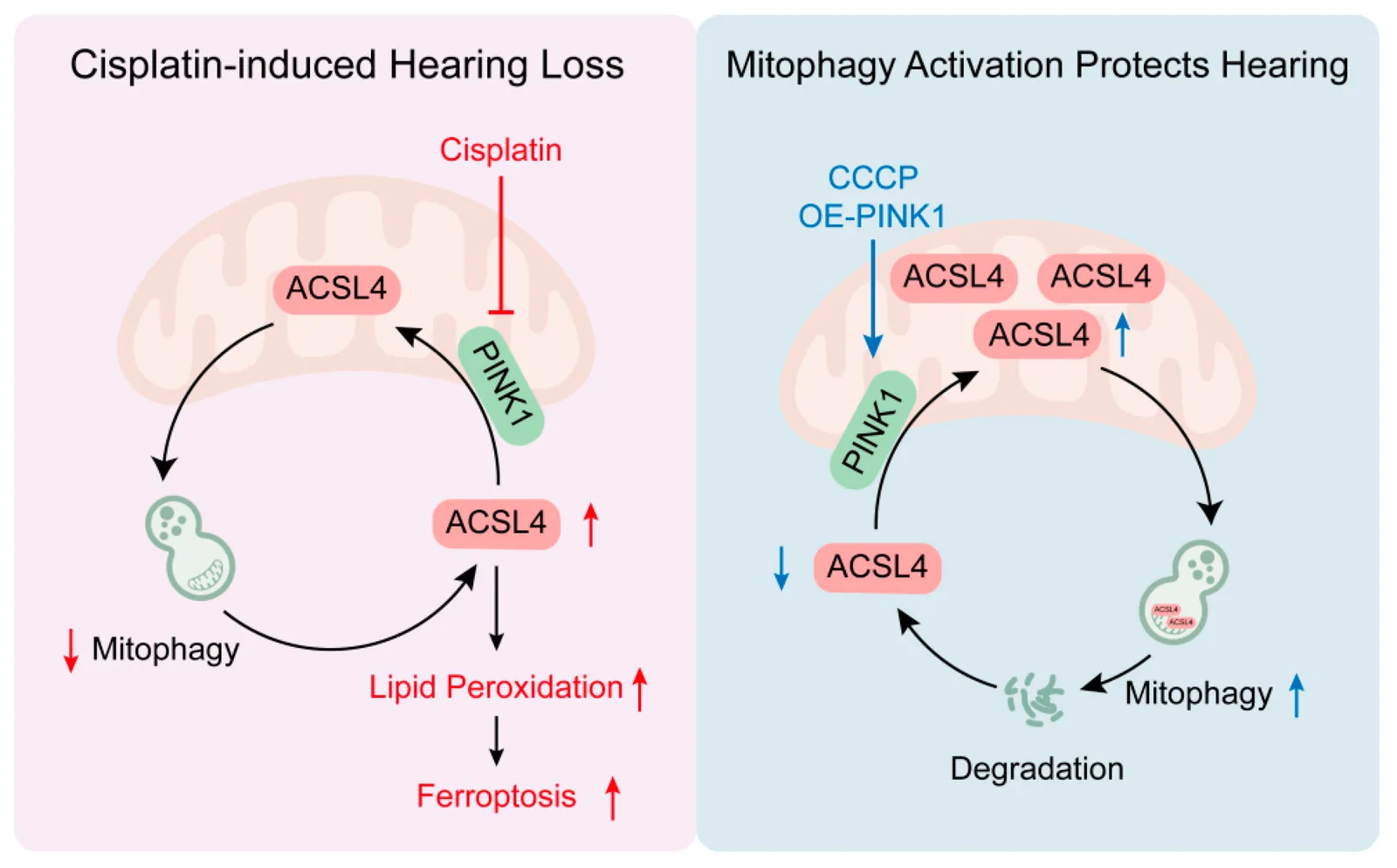
Cisplatin promotes ACSL4-mediated ferroptosis and hearing loss by inhibiting PINK1-associated mitophagy in cochlear MCs (proposed model). Cisplatin inhibits PINK1-associated mitophagy, which blocks the mitochondrial translocation of ACSL4 and leads to its cytosolic accumulation. This drives lipid peroxidation and ferroptosis, ultimately resulting in hearing loss. Conversely, activation of mitophagy (e.g., by CCCP or OE-PINK1) promotes ACSL4 mitochondrial targeting and reduces its cytosolic accumulation, potentially by facilitating its mitophagic clearance, thereby attenuating ferroptosis.

In summary, our findings support a model in which cisplatin-induced mitophagy impairment is associated with reduced mitochondrial targeting and increased cytosolic accumulation of ACSL4, thereby promoting lipid peroxidation and ferroptosis. Conversely, restoration of mitophagy by CCCP facilitates ACSL4 mitochondrial targeting and reduces its cytosolic abundance, potentially through coordinated mitochondrial translocation and mitophagic degradation. This work identifies dynamic regulation of ACSL4 subcellular localization as a critical nexus through which mitophagy governs ferroptotic cell death (Figure 10).

## 4. Discussion

In the present study, we investigated the relationship between mitophagy and ferroptosis in cisplatin-induced ototoxicity, with a particular focus on cochlear MCs. Our findings support three principal conclusions. First, cisplatin-induced injury in MCs was accompanied by mitochondrial dysfunction, impaired mitophagic activity, and ferroptotic changes. Second, pharmacological induction of mitophagy and PINK1 upregulation attenuated these ferroptosis-associated changes and improved cell viability. Third, cisplatin altered the subcellular distribution of ACSL4, whereas restoration of mitophagy increased its mitochondrial targeting and reduced its cytosolic abundance. Together, these findings support a model in which PINK1-associated mitophagy modulates cisplatin-induced ferroptotic injury, potentially through the regulation of ACSL4 subcellular distribution and clearance.

Ferroptosis has been increasingly implicated in cisplatin-induced cochlear injury. Previous studies have linked iron dysregulation and lipid peroxidation to damage in different cochlear cell populations. The forkhead box O1 (FOXO1)-NCOA4 axis promotes ferritinophagy and ferroptosis in spiral ganglion neurons, whereas modulation of the suppressor of cytokine signaling 1 (SOCS1)–NCOA4 pathway attenuates cisplatin-induced hair-cell injury [13,14]. Cisplatin-induced uric acid release from stria vascularis cells has also been reported to promote ferritinophagy and ferroptosis in hair cells [6]. In addition, disruption of GPX4–mediated antioxidant defense increases the susceptibility of cochlear hair cells and synapses to ferroptotic injury [15]. Consistent with these observations, cisplatin-treated MCs in the present study exhibited increased intracellular Fe^2+^ levels and lipid peroxidation, changes in ferroptosis-related proteins, and ferroptosis-associated mitochondrial abnormalities. The protective effects of Fer-1 further support the involvement of ferroptotic injury in cisplatin-induced MC damage. Given the close relationship between mitochondrial dysfunction and lipid peroxidation, we further investigated whether impaired mitochondrial quality control contributed to ferroptotic injury in MCs. Mitophagy is a major mitochondrial quality-control process, but its role in ferroptosis appears to depend on cellular context. In cochlear models, inhibition of mitophagy has been associated with increased cisplatin cytotoxicity and age-related hearing loss, whereas enhancement of mitophagic activity has shown protective effects [16,22,23]. Similarly, in other cisplatin-injury models, disruption of PINK1, PARK2, or BCL2-interacting protein 3 (BNIP3) aggravated ferroptosis, while mitophagy induction attenuated ferroptotic damage [24,25]. Conversely, mitophagy has also been reported to facilitate ferroptosis under certain conditions. In gastric cancer cells, Natriuretic peptide receptor 1 (NPR1)-mediated inhibition of mitophagy-dependent ferroptosis has been linked to cisplatin resistance [36]. In the present study, cisplatin suppressed PINK1-associated mitophagy, as evidenced by altered mitophagy-related protein expression and reduced mitochondria-lysosome colocalization. Both CCCP-induced mitophagy and PINK1 overexpression attenuated ferroptosis-associated changes and improved cell viability. Collectively, these results support a protective role for PINK1-associated mitophagy in cisplatin-treated MCs.

A major finding of this study is the association between mitophagy and the subcellular distribution of ACSL4. ACSL4 promotes the incorporation of polyunsaturated fatty acids into membrane phospholipids, thereby increasing the availability of substrates for lipid peroxidation and ferroptosis [28,29]. Previous studies showed that ACSL4 can localize to mitochondria-associated membranes and other subcellular compartments [30]. In cardiac microvascular endothelial cells, inhibition of ACSL4 mitochondrial translocation attenuated mitochondria-associated ferroptosis, suggesting that mitochondrial ACSL4 can promote local lipid peroxidation [31,32]. In contrast, cisplatin-treated MCs in our study showed reduced mitochondrial localization but increased cytosolic abundance of ACSL4, accompanied by enhanced lipid peroxidation and ferroptotic injury. This difference suggests that the functional consequences of ACSL4 redistribution may vary among cell types and injury conditions.

Restoration of mitophagy partially recovered ACSL4 mitochondrial localization while reducing its cytosolic abundance, without producing detectable accumulation in the mitochondrial fraction. These findings are consistent with a model in which ACSL4 is targeted to damaged mitochondria and subsequently cleared during mitophagy. Under cisplatin-induced mitophagy impairment, reduced mitochondrial targeting or clearance may allow ACSL4 to accumulate in the cytosolic fraction, thereby increasing the cellular capacity for phospholipid peroxidation. Conversely, restoration of mitophagy may reduce the pathogenic cytosolic ACSL4 pool through coordinated mitochondrial targeting and clearance. This interpretation could reconcile the increased ACSL4–Tomm20 colocalization with the absence of detectable mitochondrial ACSL4 accumulation following mitophagy induction. However, the present immunofluorescence and subcellular fractionation data do not directly demonstrate ACSL4 trafficking or degradation. Direct measurement of ACSL4 turnover and lysosomal degradation will therefore be required to validate this proposed mechanism. Notably, as a membrane-associated protein, ACSL4 may localize to mitochondria-associated membranes (MAMs) via protein–protein interactions [30]. Therefore, we speculate that cisplatin-induced mitophagy impairment triggers ferroptosis by disrupting ACSL4′s interaction with mitochondrial docking proteins, thereby blocking its translocation and leading to cytosolic accumulation. The specific proteins involved warrant further investigation.

The in vivo findings provide additional evidence for the relevance of iron-dependent injury in cisplatin ototoxicity. Iron chelators have shown protective effects in several experimental models of cochlear damage. DFO has been reported to reduce oxidative injury in cochlear explants and auditory cell models, whereas DFP protected against aminoglycoside-induced hair-cell injury and was associated with enhanced clearance of damaged mitochondria [34,35,37,38]. In the present study, DFO attenuated cisplatin-induced high-frequency hearing loss, stria vascularis edema, and blood–labyrinth barrier disruption. These effects were accompanied by increased PINK1 and decreased ACSL4 immunoreactivity in the cochlear stria vascularis. This molecular pattern was consistent with the changes observed following mitophagy induction in vitro.

Several limitations should be considered. First, the mechanistic experiments were primarily conducted in cultured rat cochlear MCs. Because cisplatin also injures hair cells, spiral ganglion neurons, and other cochlear cell populations, the generalizability of the proposed PINK1–ACSL4 relationship requires further evaluation. Second, although ACSL4 redistribution was supported by immunofluorescence and subcellular fractionation, its mitochondrial trafficking and mitophagy-dependent degradation were not directly measured. The proteins responsible for ACSL4 mitochondrial targeting also remain unknown. Finally, validation in cell-type-specific genetic models and assessment of other clinically relevant cochlear cell populations will be needed to determine the broader significance of this regulatory relationship.

## 5. Conclusions

In summary, our findings support a protective role for PINK1-associated mitophagy in cisplatin-induced ferroptotic injury. Restoration of mitophagy was associated with increased mitochondrial targeting and reduced cytosolic accumulation of ACSL4, suggesting a potential mechanism linking mitochondrial quality control to ferroptosis in cochlear MCs. The accompanying protective effects of DFO in vivo further indicate that modulation of iron-dependent injury and mitochondrial homeostasis warrants continued investigation as a strategy against cisplatin-induced hearing loss.

## Data Availability

The RNA-sequencing data generated in this study are publicly available in the NCBI Sequence Read Archive under BioProject accession number PRJNA1402639 (https://www.ncbi.nlm.nih.gov/bioproject/PRJNA1402639, accessed on 10 September 2026). The other data supporting the findings of this study are available within the article and its Supplementary Materials or from the corresponding author upon reasonable request.

## Supplementary Materials

The following supporting information can be downloaded at https://www.mdpi.com/article/10.3390/cells15181657/s1, Table S1: Primary Antibody Details; Table S2: Primer sequences used for qPCR.

## Abbreviations

The following abbreviations are used in this manuscript:

ABR	auditory brainstem response
ACSL4	Acyl-CoA synthetase long-chain family member 4
BCA	bicinchoninic acid
BLB	blood–labyrinth barrier
CCK-8	Cell Counting Kit-8
CCCP	carbonyl cyanide m-chlorophenyl hydrazone
Cis	cisplatin
DAB	3,3′-diaminobenzidine
DAPI	4′,6-diamidino-2-phenylindole
DEG	differentially expressed gene
DFO	deferoxamine
DFP	deferiprone
ECL	enhanced chemiluminescence
FDR	false discovery rate
Fer-1	Ferrostatin-1
FOXO1	forkhead box O1
FSP1	ferroptosis suppressor protein 1
GO	Gene Ontology
GPX4	glutathione peroxidase 4
GSEA	gene set enrichment analysis
H&E	hematoxylin and eosin
HBSS	Hanks’ balanced salt solution
HRP	horseradish peroxidase
IHC	immunohistochemistry
KEGG	Kyoto Encyclopedia of Genes and Genomes
LC3	Microtubule-associated protein 1 light chain 3
LDH	lactate dehydrogenase
MAM	Mitochondria-associated membrane
MC	marginal cell
MMP	mitochondrial membrane potential
NCOA4	nuclear receptor coactivator 4
OE-PINK1	PINK1 overexpression
PARK2	parkin RBR E3 ubiquitin protein ligase
PBS	Phosphate-buffered saline
PINK1	PTEN-induced putative kinase 1
PVDF	polyvinylidene difluoride
qPCR	quantitative polymerase chain reaction
RNA-seq	RNA sequencing
ROS	reactive oxygen species
SD	Sprague–Dawley
SDS-PAGE	sodium dodecyl sulfate–polyacrylamide gel electrophoresis
SGN	spiral ganglion neuron
SV	stria vascularis
TEM	transmission electron microscopy
Tomm20	translocase of outer mitochondrial membrane 20
TPM	transcripts per million
