# Supplementary material for: PINK1–Associated Mitophagy Protects Against Cisplatin–Induced Ototoxicity by Regulating the Subcellular Distribution of ACSL4
Source: Cells. 2026 Sep 14;15(18):1657. doi: 10.3390/cells15181657 (PMC13605962; doi:10.3390/cells15181657)
Supplement: Supplementary file 1 [file cells-15-01657-s001.zip › cells-4540376-supplementary.pdf]

Supplementary Materials

Table S1. Primary antibody details.

Primary Antibody	Application
β-actin	WB（1:2000, GB15003, Servicebio）
PINK1	WB（1:2000, 23274-1-AP, Proteintech）
	IHC（1:50, 23274-1-AP, Proteintech）
Parkin	WB（1:1000, T56641S, Abmart）
p62	WB（1:2000, 23214T, CST）
LC3	WB（1:2000, 43566T, CST）
Tomm20	WB（1:2000, 42406T, CST）
	IF（1:200, 42406T, CST）
GPX4	WB（1:2000, 32506, SAB）
ACSL4	WB（1:2000, 22401-1-AP, Proteintech）
	IF（1:200, 38493T, CST）
	IHC（1:2000, 22401-1-AP, Proteintech）

PINK1: PTEN-induced putative kinase 1; GPX4: glutathione peroxidase 4; ACSL4: acyl-CoA synthetase long-chain family member 4.

Table S2. Primer sequences used for qPCR in primary rat cochlear marginal cells.

Primer Name	Forward Primer Sequence (5' – 3')	Reverse Primer Sequence (5' – 3')
β-actin	ACCCACACTGTGCCCATCTAC	TCGGTGAGGATCTTCATGAGGTA
GPX4	GCCGAGTGTGGTTTACGAATC	GCATCGTCCCCATTTACACAG
NCOA4	TCTGGAGAAGCCTGTGAATGG	GCTGGTTACCCTGACAGTTGG
Ferritin	CTGGAACTTCACAAACTGGCTAC	GGTGTGCTTGTCAAAGAGATATTC
FSP1	ATCAAGGTGGAAACGGACAAG	GCCAGCCTACTCTCAAACGC
ACSL4	AGTACAACTTTCCGCTTGTG	AAGCCTCAGACTCATTTAATCC
PINK1	TTCTTCCGCCAGTCGGTGGC	CTGCTTCTCCTCGATCAGCC

NCOA4: nuclear receptor coactivator 4; FSP1: ferroptosis suppressor protein 1.
